# Supplementary material for: Genome-Wide Identification and Characterization of CDPK Gene Family in Cultivated Peanut (Arachis hypogaea L.) Reveal Their Potential Roles in Response to Ca Deficiency
Source: Cells. 2023 Nov 21;12(23):2676. doi: 10.3390/cells12232676 (PMC10705679; doi:10.3390/cells12232676)
Supplement: Supplementary file 1 [file cells-12-02676-s001.zip › Supplementary Figures.pdf]

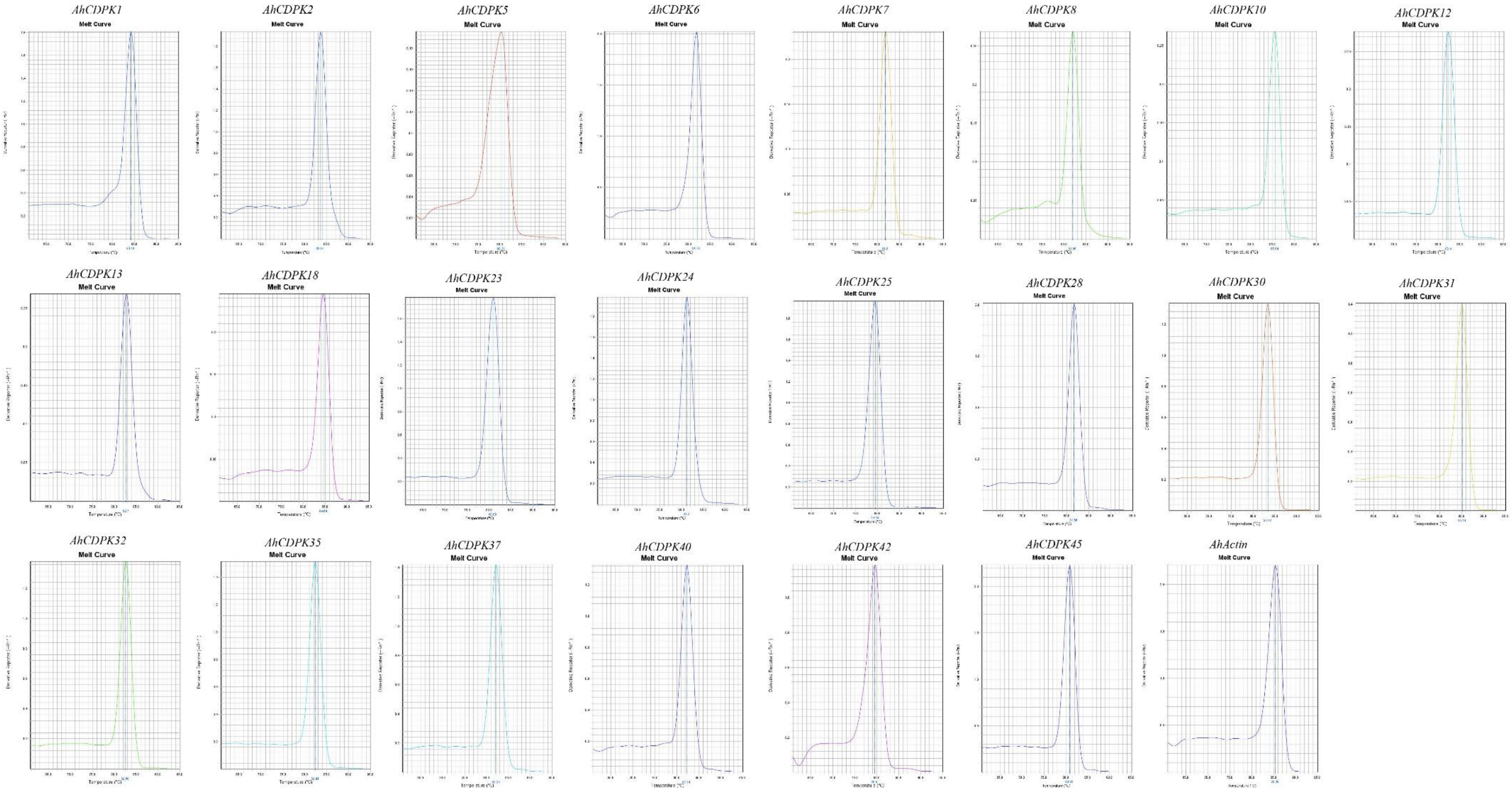

Figure S1. Melt-curve analysis of the selected *AhCDPK* genes and *AhActin* for qRT-PCR analysis.

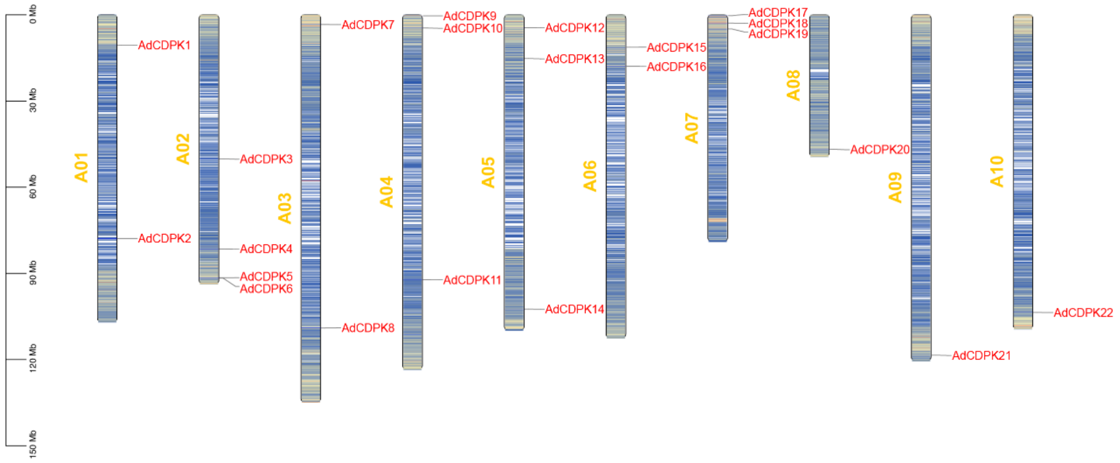

Figure S2. Chromosomal distribution of the *CDPK* genes in *A.duranensis*.

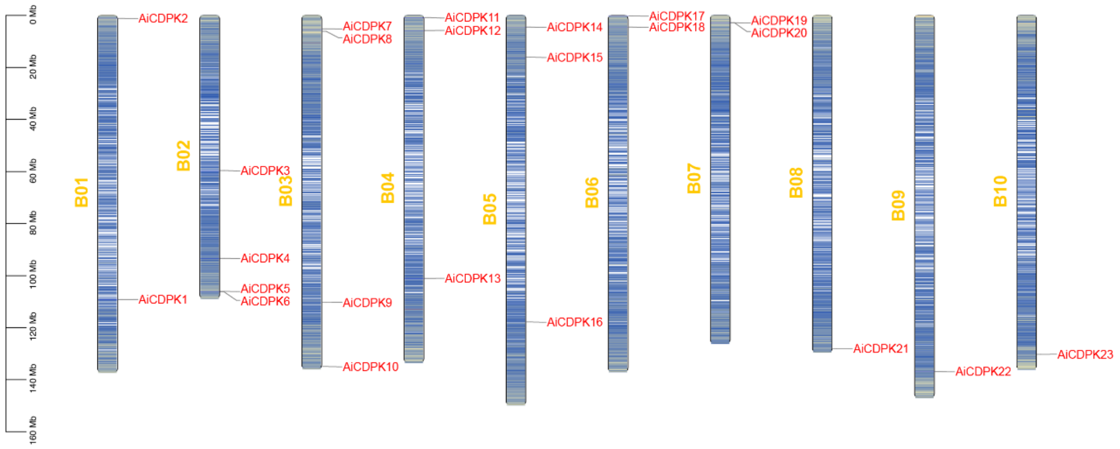

Figure S3. Chromosomal distribution of the *CDPK* genes in *A.ipaensis*.

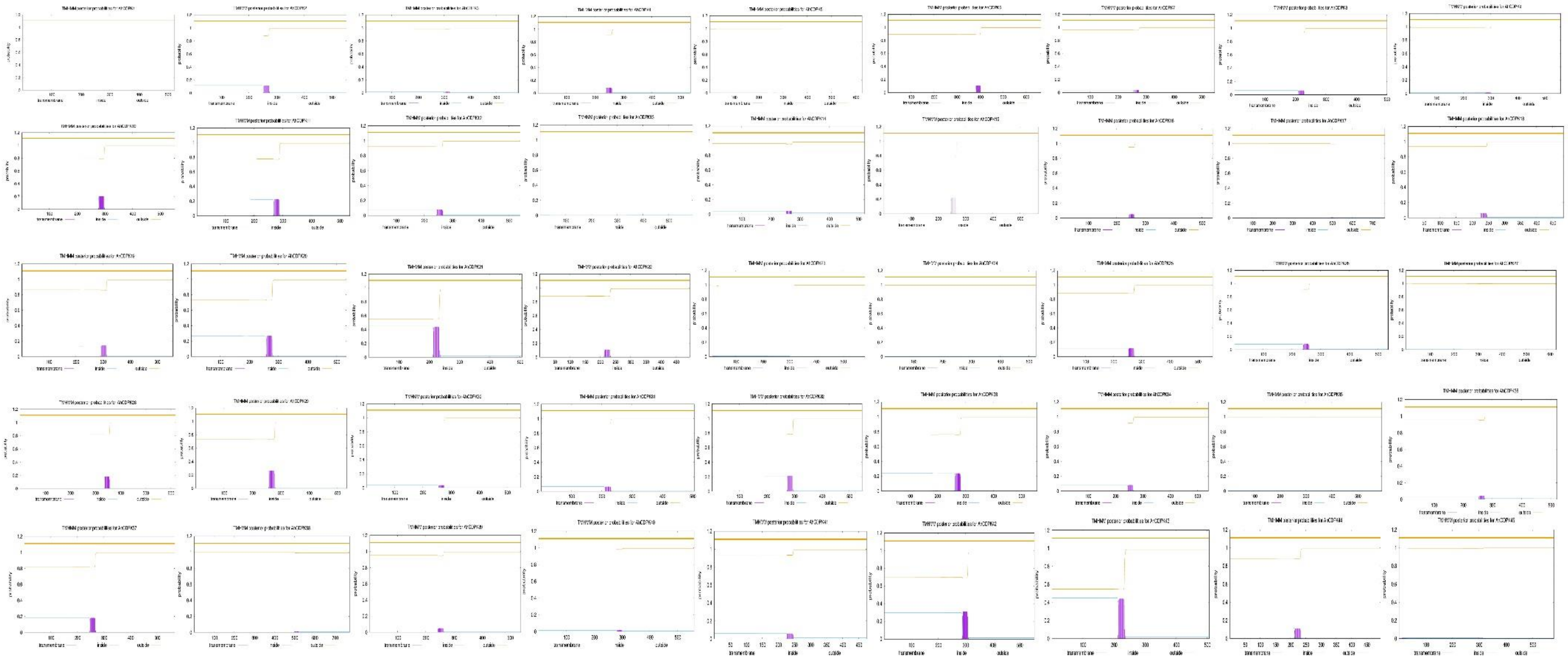

Figure S4. Prediction of transmembrane structure of AhCDPKs.

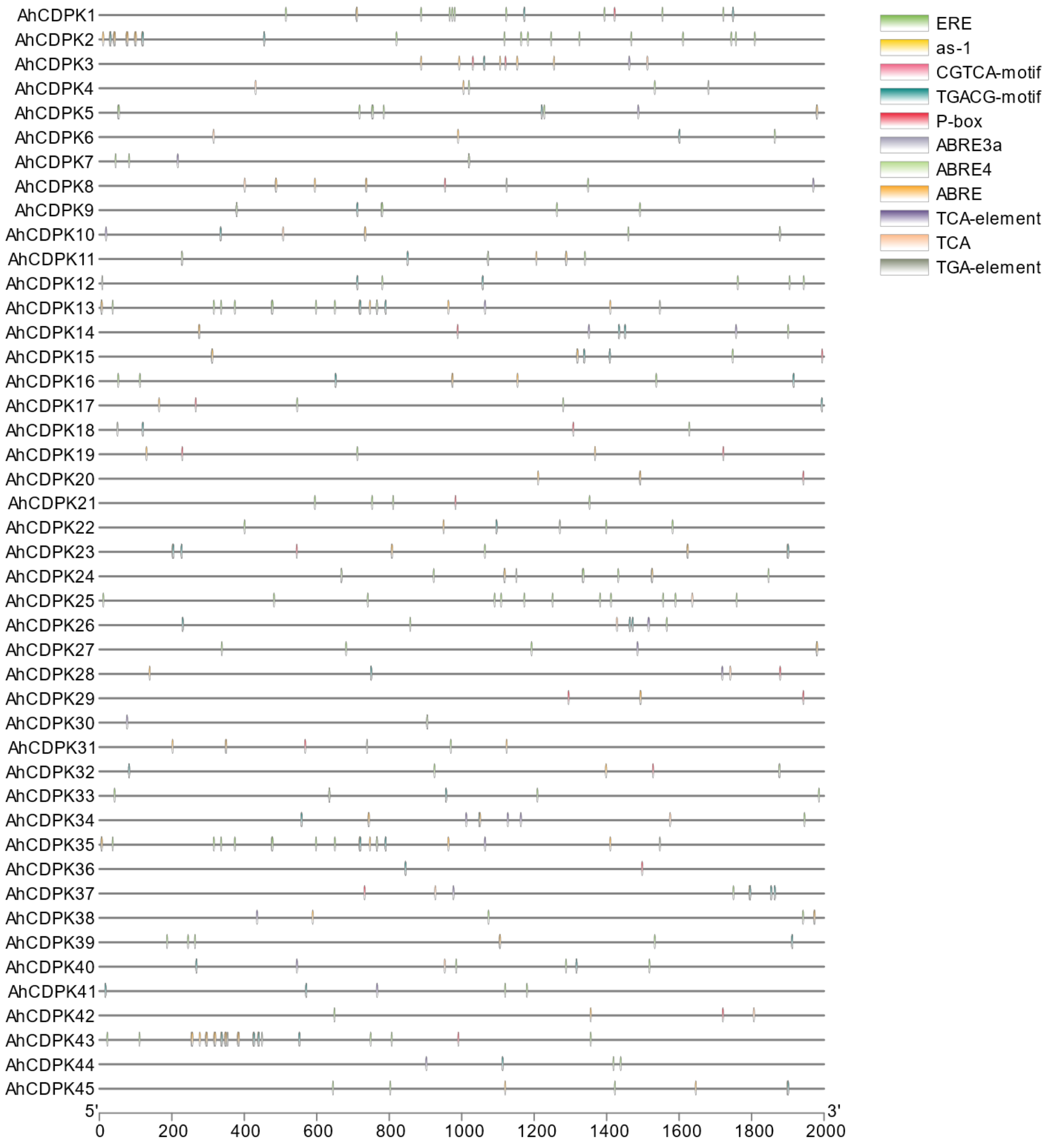

Figure S5. The positional distribution of phytohormone responsive cis-regulatory elements on promoters is shown as vertical bars with different colors.

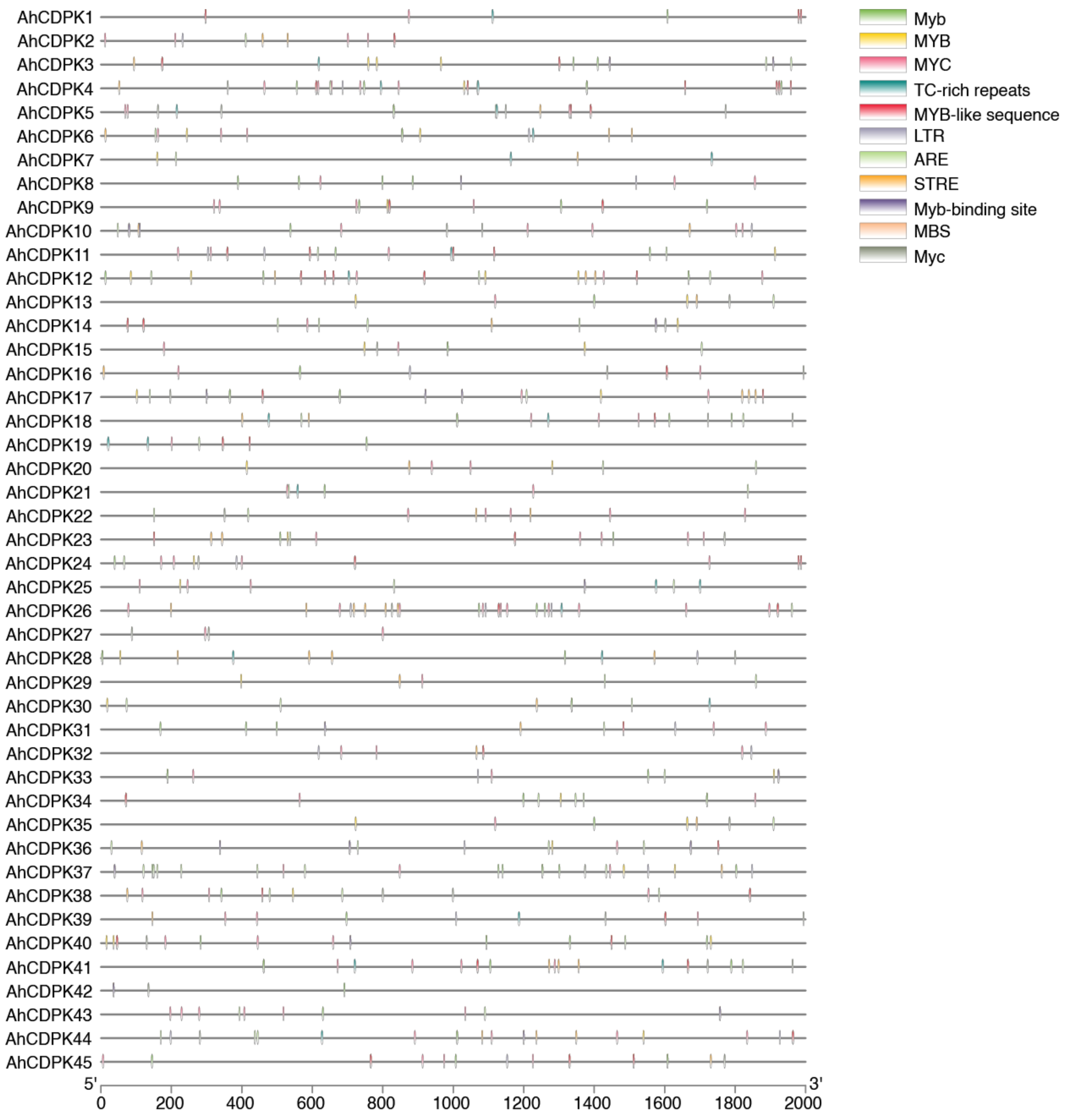

Figure S6. The positional distribution of abiotic stress cis-regulatory elements on promoters is shown as vertical bars with different colors.

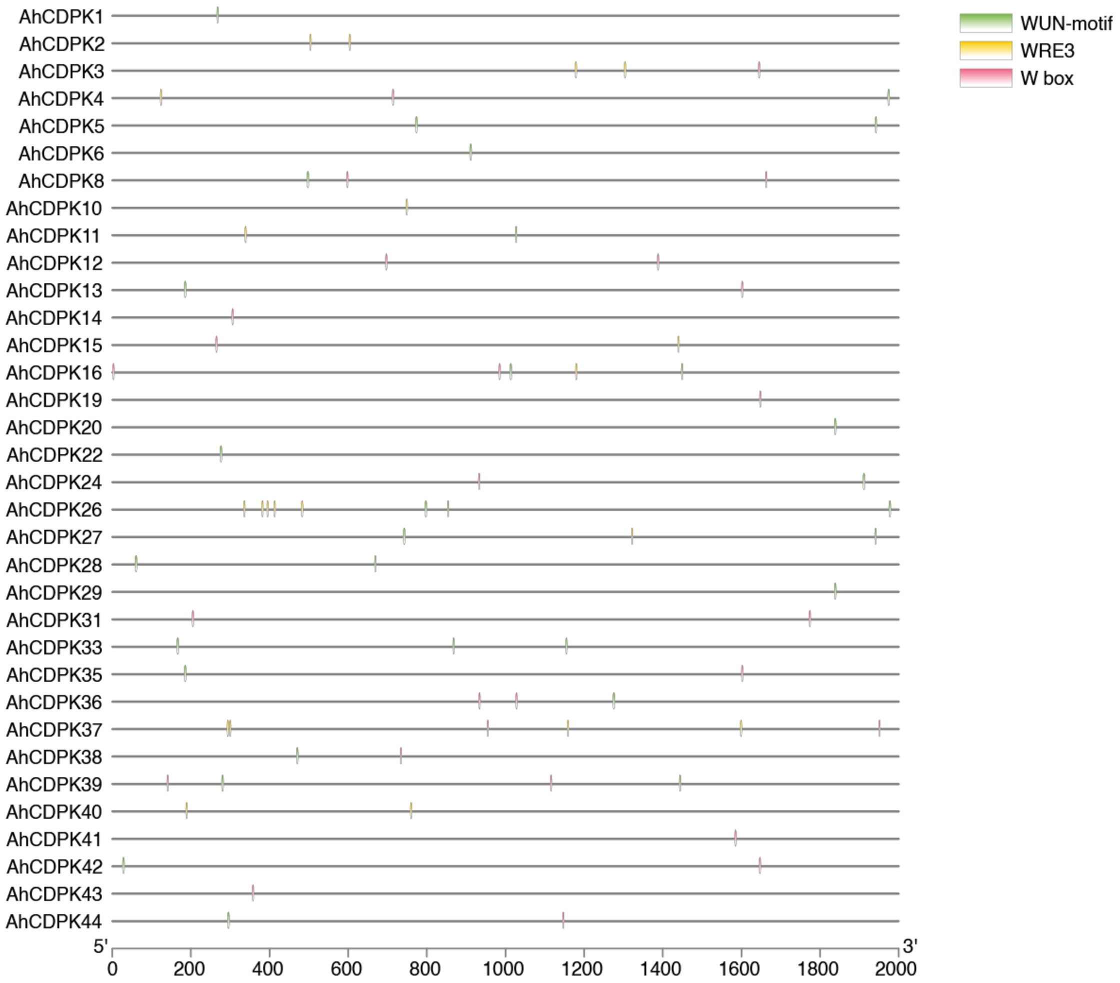

Figure S7. The positional distribution of biotic stress cis-regulatory elements on promoters is shown as vertical bars with different colors.

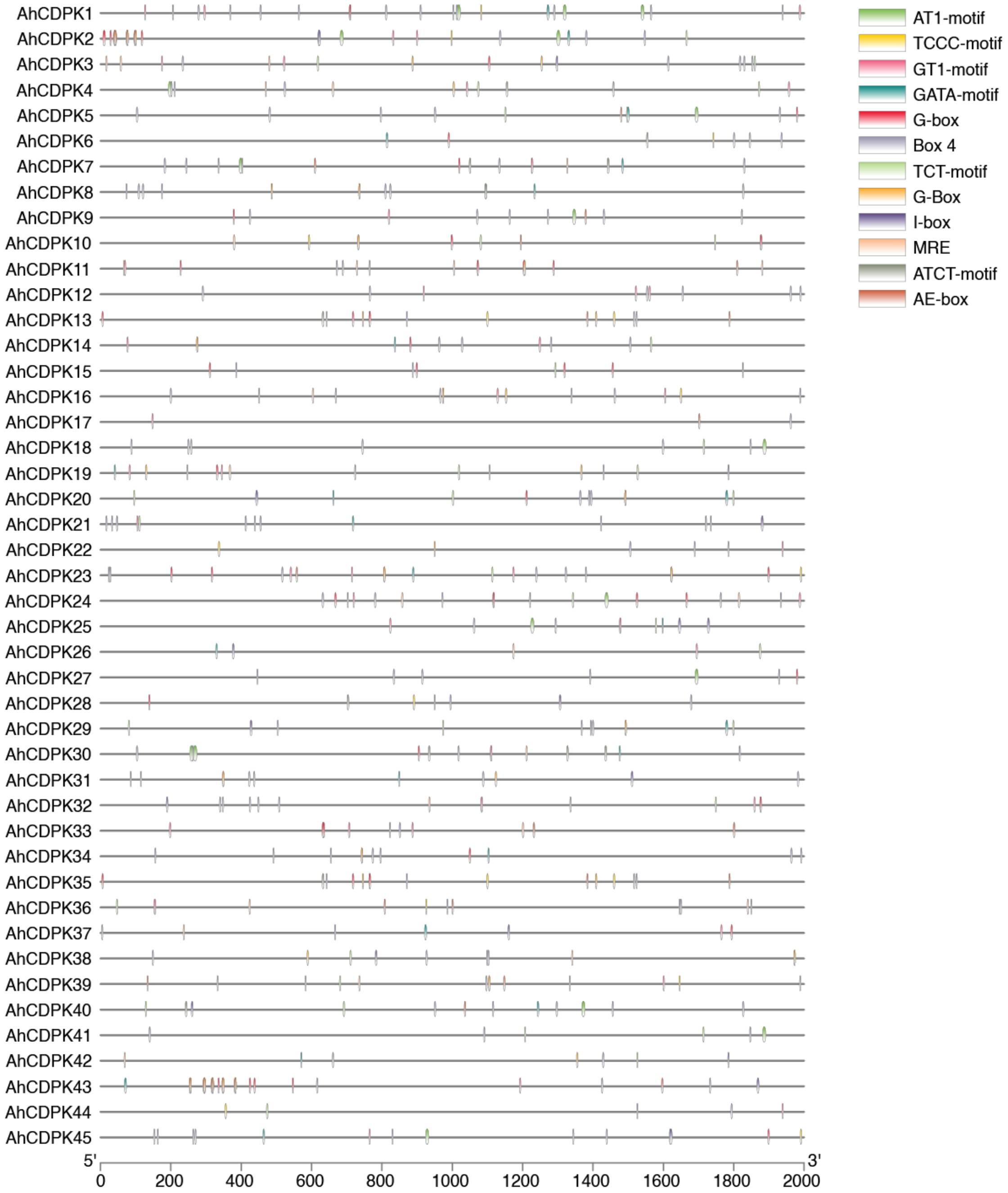

Figure S8. The positional distribution of light responsive cis-regulatory elements on promoters is shown as vertical bars with different colors.

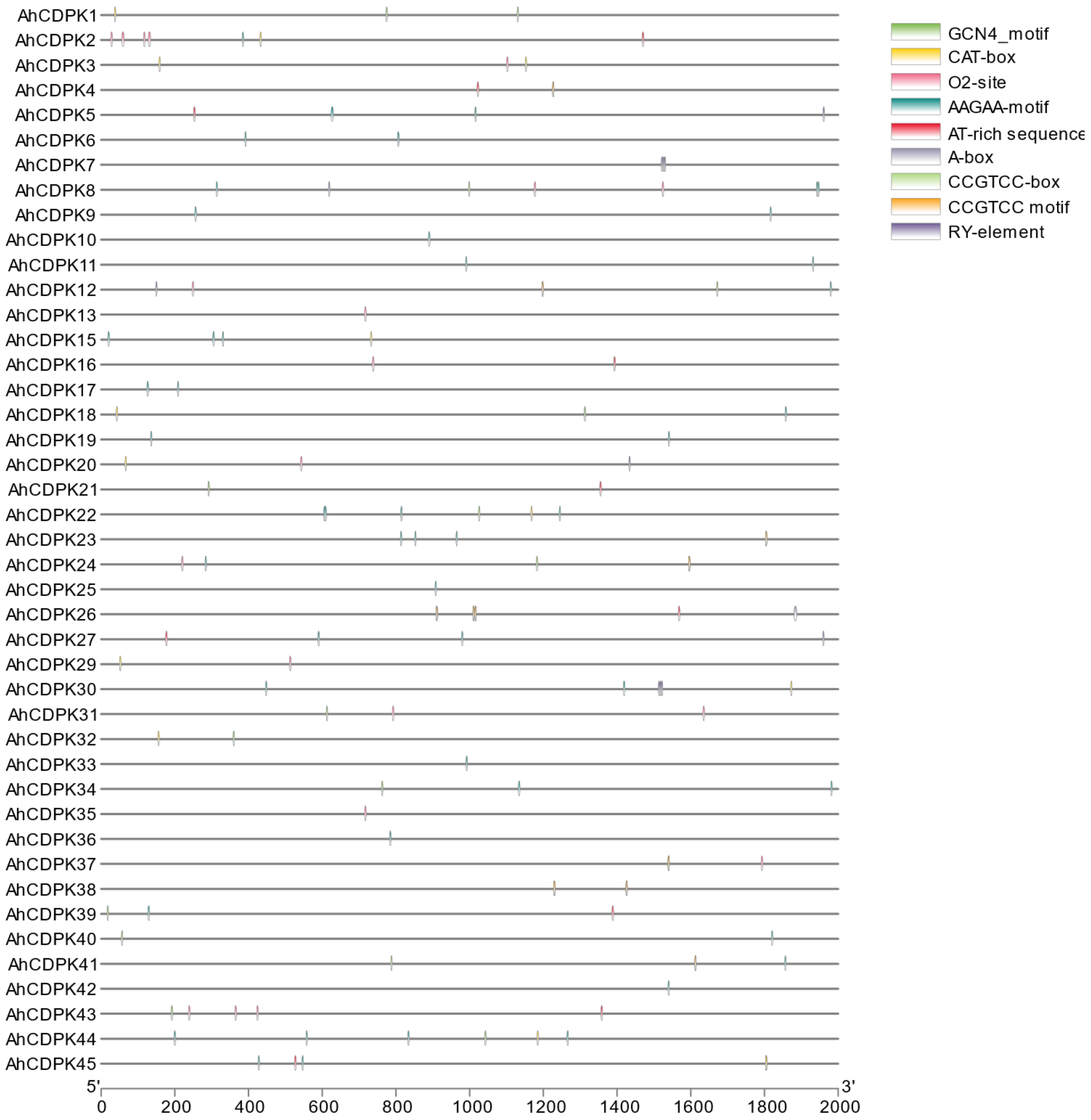

Figure S9. The positional distribution of plant growth and development cis-regulatory elements on promoters is shown as vertical bars with different colors.

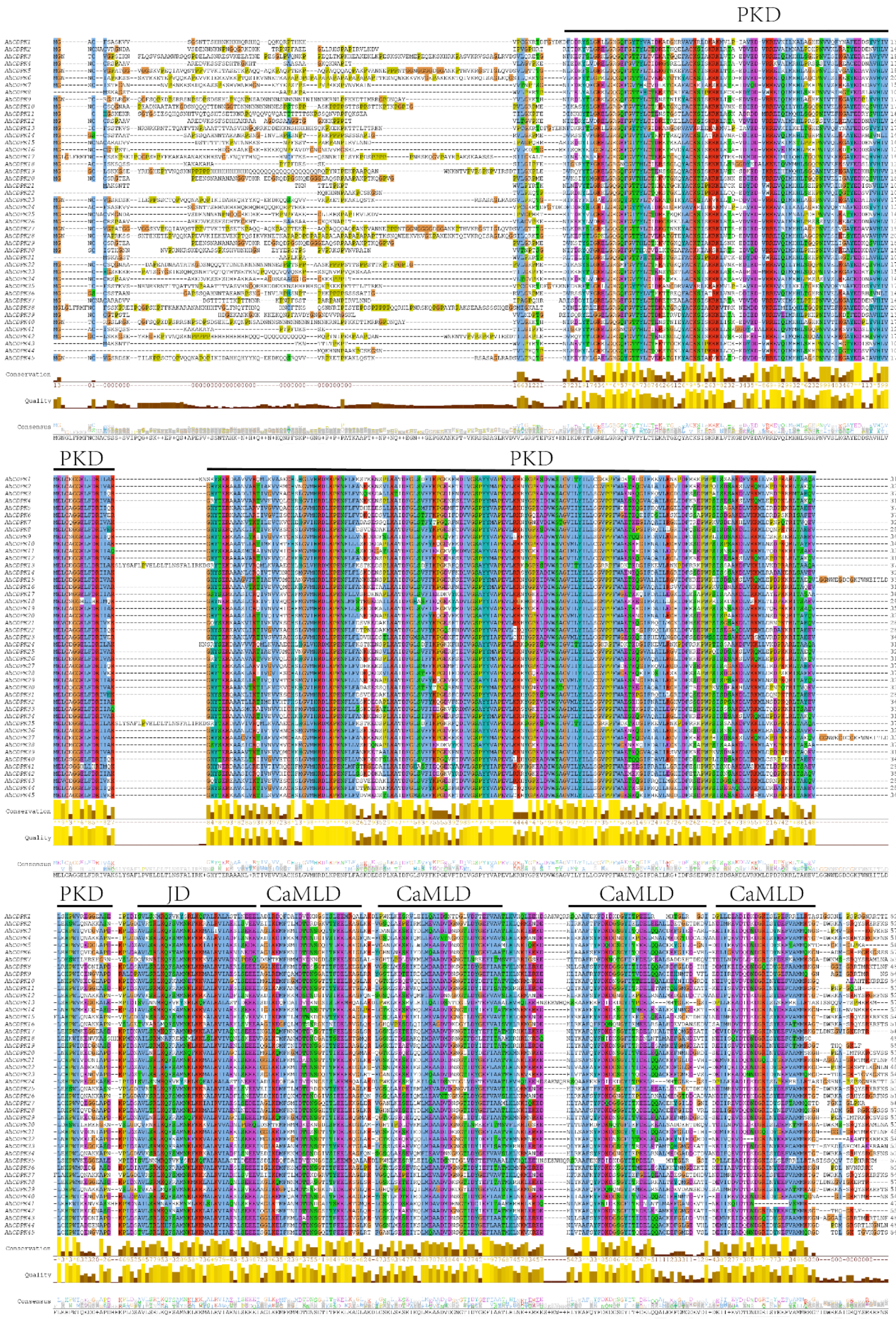

Figure S10. Multiple sequence alignment of conserved domain of AhCDPK proteins. PKD, JD and CaMLD were indicated by black horizontal line.
